# Study of the electromechanical coupling process before the 2020 *M*_s_ 6.4 Yutian, China earthquake

**DOI:** 10.1038/s41598-022-22532-2

**Published:** 2022-10-21

**Authors:** Chen Yu, Shuyan Wang, Huaizhong Yu

**Affiliations:** 1 China Earthquake Networks Center, Beijing, 100045 China; 2 Academy of Electronics and Information Technology, China Electronics Technology Group Corporation, Beijing, 100041 China

**Keywords:** Natural hazards, Solid Earth sciences

## Abstract

The observation of electromechanical coupling might be used as an important tool to detect pre-seismic changes associated with the preparation of earthquakes. This paper attempts to study the electromechanical coupling process before the large earthquakes by using the load/unload response ratio (LURR) approach in which the geo-electric data and Benioff strain of small earthquakes were adopted as the data input. The variation of Coulomb failure stress induced by earth tides on the fault surface of the mainshock is applied to differentiate the loading and unloading stages. Using this technique, we test the geo-electric data recorded at the Hotan observatory near the epicenter of 2020 Yutian *M*_s_ 6.4 earthquake. Results show that the LURR time sequence fluctuated around 1.0 for many years and reached significant high peaks at the beginning of 2020. More importantly, this evolution correlates well with the LURR time series calculated by using the Benioff strain of small earthquakes within the circular region of 300 km radius centered at the epicenter. The underlying physics of the changes should be caused by the fluid infiltration derived from pre-seismic rock dilatancy. The corresponding volume variations in the crust could be found in the geophysical observation time series in the same neighborhoods.

## Introduction

In recent decades, some geophysical observation data have recorded the abnormal variations of electromagnetic field in the seismogenic region prior to earthquakes^1,2^. Since Sobolev in 1975^2^ reported the abnormal change of ground electric current before Kamchatka earthquake, VAN group from Greece also has carried out a series of observations and studies on the abnormal variation of geo-electric field^3^. Moreover, they reported that the Seismic Electric Signal (SES) has been extracted from the continuous geo-electric field data by them, which triggered a series of discussions in the field of earthquake prediction^4^. In order to explain the electric field changes prior to earthquakes, scientists have proposed many hypotheses, such as electrokinetic effect, seismoelectric effect, piezoelectric effect, etc., but there is still no unified view^5,6^.

Presently, the observations of pre-seismic anomalies are mostly obtained by using the statistical approach from the observation of the original curve^7^. Corwin and Morrison^8^ investigated the medium-to-short-term pre-seismic variations in electric field (self-potential) preceding two earthquakes in central California. Zhao and Qian^9^ detected the impending-earthquake precursors in geo-electric data before the Tangshan main shock of 1976 in China. Sarlis^10^ analyzed the statistical significance of earth’s electric field variations in Greece and Japan. From these studies, however, it is difficult to construct the physical correlation between the geo-electric anomalies and earthquake.

**Figure 1:**
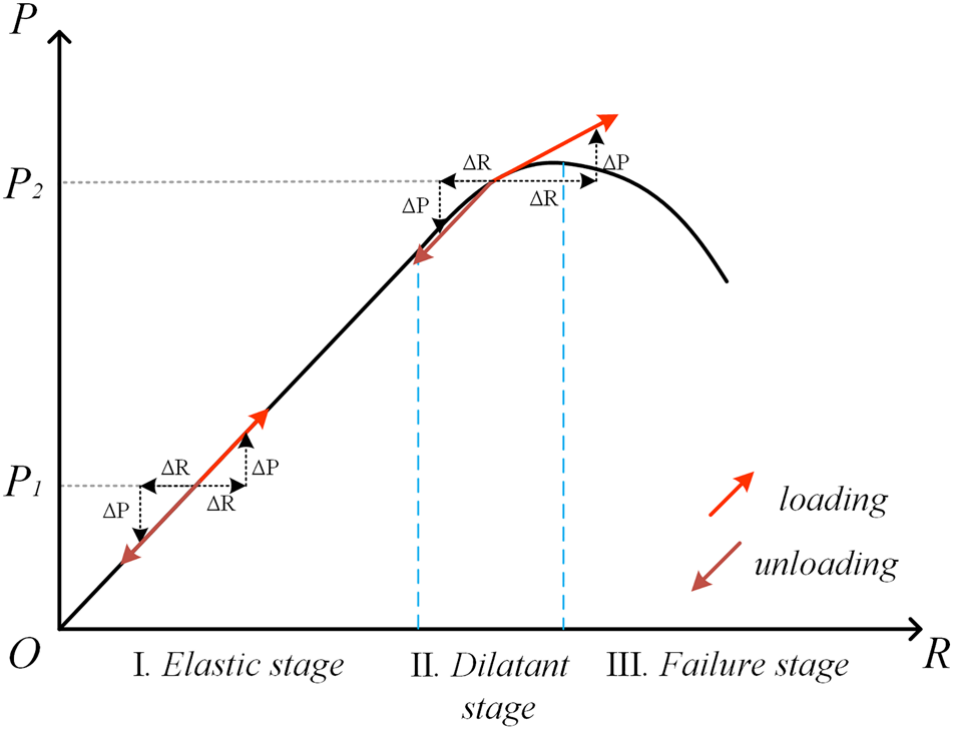
Schematic diagram of the rock constitutive relationship. *P* and *R* represent, respectively, the load and response. At the point P_1_ in stage I, the response (ΔR) to the small changes of ΔP in the loading and unloading are almost the same. At the stage II (e.g. point P_2_), the response to loading is significantly greater than unloading, and the stage III is characterized with macroscopic damage.

In recent years, Yu et al.^11^ have found that the Load/unloading response ratio (LURR) might be applied to explore nonlinear mechanical process associated with earthquake preparation via groundwater level and the pre-seismic changes were explained by dilatancy. This may provide an opportunity for detect pre-seismic electric field changes. The LURR approach was firstly devised by Yin et al.^12^ in 1995 and widely applied to retrospective earthquake prediction practices^13–15^. The variation of LURR reflects the dynamical changes of stress state of a rock system (Fig. 1). When the rock is in the elastic stage, the responses to the loading and unloading are nearly the same, and LURR = 1.0; when the rock is stressed to the dilatant state, the responses are quite different, generally, LURR > 1.0. The LURR value is therefore served as a useful tool to analyze the criticality of earthquakes, and, the anomalous increases in the time series of LURR preceding a large earthquake has been reported by many publications^16,17^.

In fact, with the development of digital signal processing techniques, recent studies have found that changes in geo-electric field observations are also affected by earth tides^18^. The fractures and cracks caused by pre-earthquake stress accumulation can cause rock dilatancy, and the increase in crustal volume may lead to changes in fluid migration activities, which can lead to changes in the geo-electric field^19^. Hence, the geo-electric field observation data may also be served as the loading/ unloading response for LURR calculation. On the other hand, in the traditional LURR calculation, the Benioff strain release during loading and unloading periods is usually adopted as the data input^20,21^. If we can establish the correlation between the LURR time series obtained by using the Benioff strain and geo-electric data, the explanation for the pre-seismic geo-electric field changes may be obtained from the electromechanical coupling process.

In this paper, we attempt to detect the pre-seismic electromechanical coupling process in the source area by applying the LURR method to identify the difference in geo-electric data and seismicity during loading and unloading caused by earth tides. The rule for judgment of loading and unloading period follows the theory of Coulomb failure stress change given by Yin et al.^22^. To verify the effectiveness of this approach, the 2020 *M*_s_ 6.4 Yutian, China earthquake was taken as the example. This paper is divided into seven sections. In addition to the introduction section, “Geologic setting and environment of observatory” section introduces the tectonic environment and observation conditions of the source area; “Method” section is about the method for LURR calculation of the geo-electric field; “Application and interpretation” section show the application of this approach to seismic data and some interpretations, and in “Discussion” and “Conclusion” section we would like to give some discussions and final conclusions.

## Geologic setting and environment of observatory

### Tectonic setting

**Figure 2:**
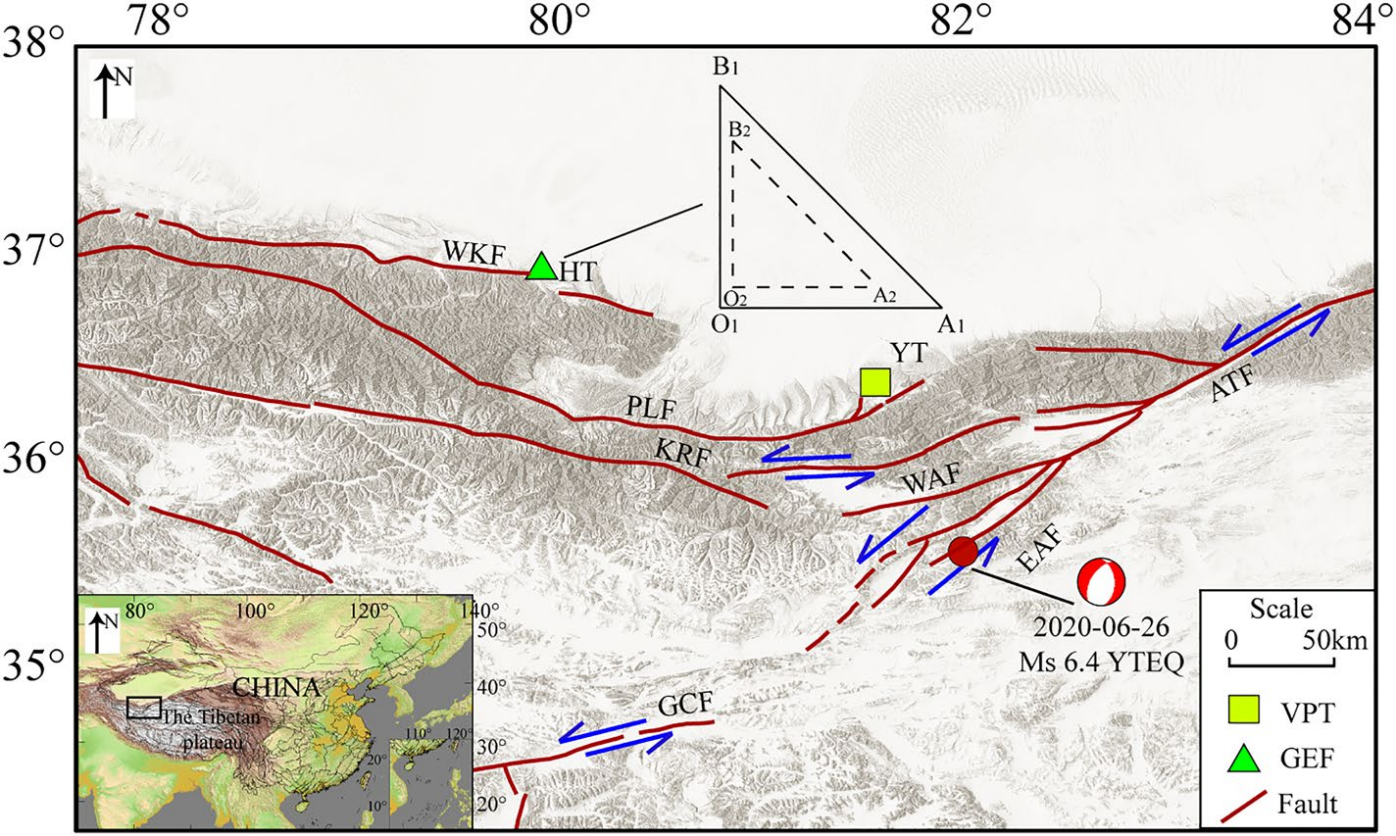
Main faults and geophysical observatories near the *M*_s_ 6.4 Yutian earthquake that occurred in 2020. Red lines indicate faults, including West Kunlun fault (WKF), Pulu fault (PLF), Karakax fault (KRF), Altun Tagh fault (ATF), west branch of Ashikule fault (WAF), east branch of Ashikule fault (EAF) and GozhaCo fault (GCF). Blue arrows indicate the relative motion of some faults. The focal mechanism represents the Yutian (YTEQ) earthquake. Square is locations of vertical pendulum tilt (VPT) in Yutian (YT) station. Triangle is locations of geo-electric field (GEF) in Hotan (HT) station. Top middle: the schematic damage of the electrode layout employed in the geo-electric field measurements. O_1_A_1_ = O_1_B_1_ = 225 m; O_2_A_2_ = O_2_B_2_ = 150 m; A_1_B_1_ = 318 m; A_2_B_2_ = 212 m. The figure was generated by using the Generic Mapping Tools (GMT) 4 (https://www.generic-mapping-tools.org/download/, accessed on 27 September 2022).

According to the China Earthquake Networks Center (CENC), an *M*_s_ 6.4 earthquake occurred in Yutian County in Hotan Prefecture of the Xinjiang Uygur Autonomous Region at 5:30 a.m. on June 26, 2020, with its epicenter at 35.33°N and 82.33°E and at a depth of 10 km (https://www.cenc.ac.cn/cenc/dzxx/362678/index.html). The epicenter was located at the eastern end of the West Kunlun Orogenic belt at the junction of the Northwest Tibetan Plateau and Tarim Basin near the southwest end of the Altun Tagh fault zone, which is the east branch of Ashikule fault (Fig. 2).

The Indian plate subducts in a northward extrusion toward the Eurasian plate, and the ancient rock mass pushes under the block of Tarim Basin causing crustal shortening and uplift of the Qinghai–Tibet Plateau. The lower crust material is dragged eastward and this causes the formation of internal east–west tension, a south–north compression stress environment, and movement from the east to the extrusion, resulting in inhomogeneity in the plateau. Strike-slip faults with a dominant direction of thousands of kilometers are caused, resulting in several fault zones such as the Altun Tagh, Kunlun, and Karakax^23,24^. These fault zones mainly consist of left-lateral strike-slip faults. However, in the western margin of the plateau, the Indian plate has subducted under the Eurasian plate in the Pamir area, and it has reached the central plains in terms of earthquake depth. Therefore, the NNW-trending Karakoram fault is a dextral strike-slip fault.

### Hotan geo-electric field observatory

Hotan observatory is located at the northern foot of the Kunlun Mountains and the southern edge of the Taklimakan Desert, which can provide geophysical observation data for the study of the tectonic movement of the Qinghai-Tibet block. A ZD9A-II geo-electric field monitoring system,with an orthogonal “L” shape array including three direction (N–S, E–W and N–W), is installed at the observatory. Each measurement direction includes long and short distance electrodes (current electrode O_1_,O_2_ and potential electrodes A_1_, A_2_, B_1_, B_2_), so Hotan observatory can produce six channels of observation data. The specific length of each distance electrodes is shown in Table 1. Hotan Station is located far away from the urban area and less disturbed by human activities. Therefore, the observed data are real and reliable, providing valuable information for us to analyze the geoelectric field LURR anomaly evolution before the Yutian earthquake.

**Table 1:** Detailed information of the Hotan Geo-electric field Observatory.

Station name	Epocentral distance (km)	Location (Lat/Lon)	Start time	Sampling frequency	Instrument	Resolution
Hotan	290	37.07/79.92	20070501	1-min	ZD9A-II	10 μV

## Method

LURR is an important discovery for an understanding of the regularity of crustal medium destruction in the focal area^12,25^. Previous studies have found that LURR time series have obvious anomalies in months to years before large earthquakes. When the crustal medium is in a stable state, the LURR value fluctuates around 1.0. When the region enters the damage stage, the LURR ratio will be greater than 1.0, indicating that the possibility of earthquakes increases. This phenomenon can be used as an important precursory to the occurrence of earthquakes.

For the prediction of earthquakes, any geophysical quantity that can reflect the medium instability process in the seismogenic area can be used as the response quantity, and the loading and unloading process can be judged according to the Coulomb rupture stress variation (ΔCFS) caused by solar and lunar tidal forces on the seismic fracture plane. We used Benioff strain and Hotan electric field observation data as response variables to calculate LURR anomaly evolution characteristics and regional stress field variation before the occurrence of the earthquake in the source region, respectively.

Assuming *P* and *R* represent load and response respectively, the response rate X can be rewritten as:

1 $$ X = \mathop {lim}\limits_{{{\Delta }P \to o}} \frac{{{\Delta }R}}{{{\Delta }P}} $$

where Δ*R* represents the change in response *R* caused by the load variable Δ*P*. When the strength a medium to withstand is much greater than the load, $X_{ + } \approx X_{ - }$, LURR ≈ 1; when the medium is close to destruction, $X_{ + } > X_{ - }$, LURR > 1. The above method can be used to judge whether the medium is in a stable stage.

The traditional LURR based on seismic energy* E* and its related parameters as response quantity as follows:

2 $$ Y_{m} = \frac{{\left( {\mathop \sum \nolimits_{i = 1}^{N + } E_{i}^{m} } \right)}}{{\left( {\mathop \sum \nolimits_{i = 1}^{N + } E_{i}^{m} } \right)}} $$

where *E*_*i*_ represents seismic energy, "+" and "−" represent loading and unloading respectively. *m* can be 0, 1/3, 1/2, 2/3, or 1. When *m* = 1, *E*^m^ represents seismic energy. When *m* = 1/3 or 2/3, *E*^m^ represents the linear and plane scales of the seismogenic region, respectively. When *m* = 1/2, *E*^m^ represents the Benioff strain. When *m* = 0, the *Y* value corresponds to the number of earthquakes occurring during the loading and unloading process.

Since the geo-electric field observation data are vector observations, the average value of the absolute values of electromagnetic observation data within a period of time is taken as the response quantity, and the LURR is defined as:

3 $$ Y_{m} = \frac{{\frac{{\left( {\mathop \sum \nolimits_{i = 1}^{N + } \left| {E_{i} } \right|} \right)}}{{\left( {N_{ + } } \right)}}}}{{\frac{{\left( {\mathop \sum \nolimits_{i = 1}^{N - } \left| {E_{i} } \right|} \right)}}{{\left( {N_{ - } } \right)}}}} $$

In Eq. (3), *E*_*i*_ represents the geo-electric field data with a period of 8 ~ 24 h in the time period. *N*_+_ and *N*_*−*_ represent the number of geo-electric data in the loading and unloading stage in a calculation time window respectively. To avoid the strong fluctuation of LURR time series caused by too few earthquakes, the calculation time window usually contains multiple loading and unloading cycles.

## Application and interpretation

**Figure 3:**
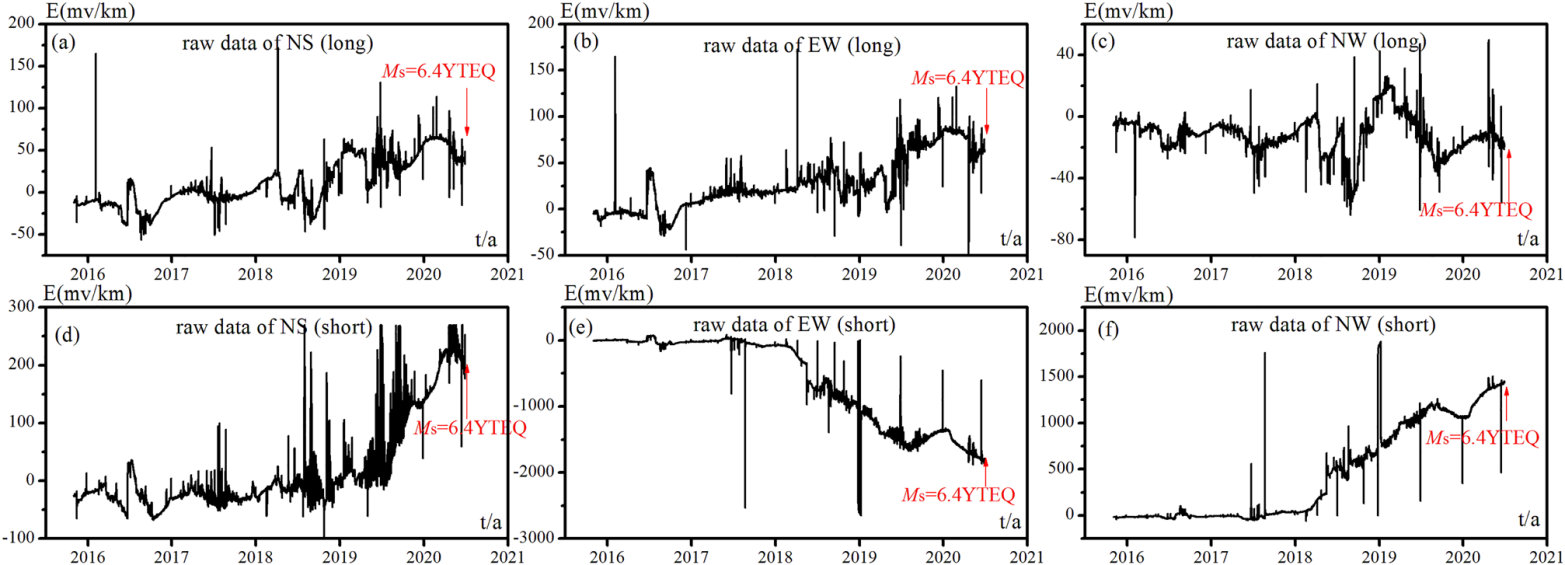
Raw data of geo-electric field for LURR calculation, with the time of earthquake in each of the maps indicated by the red vertical arrows.

**Figure 4:**
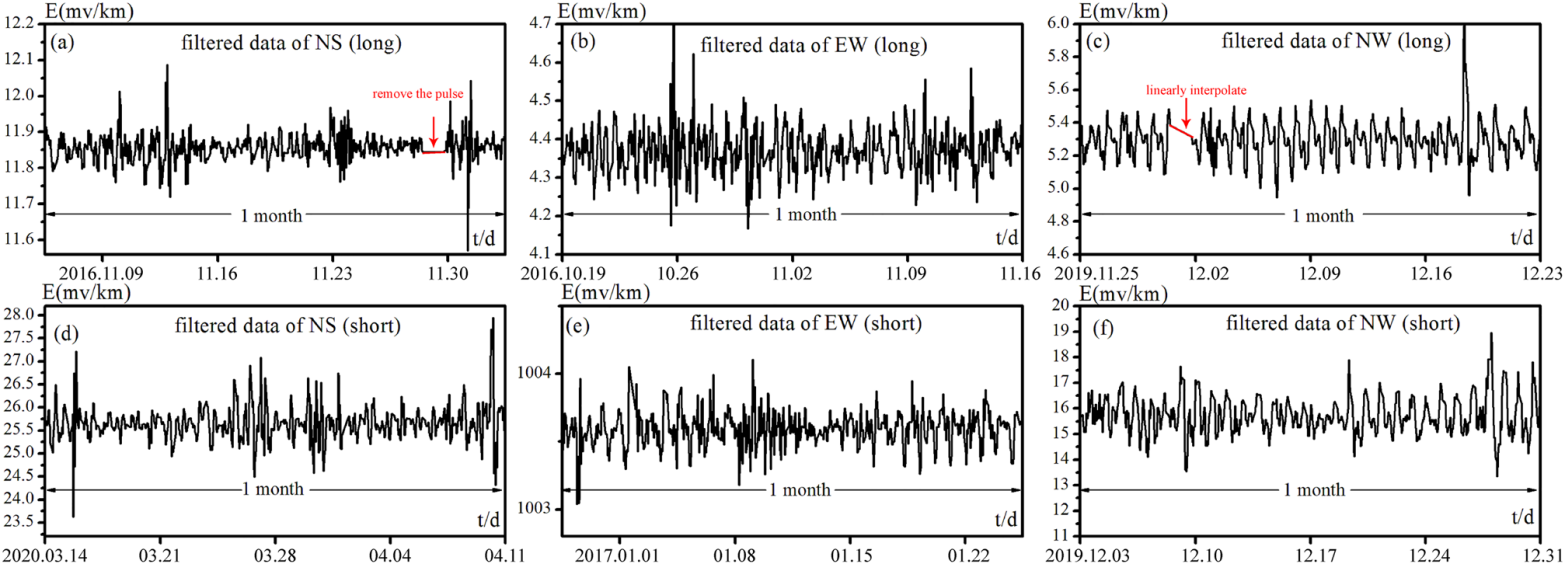
Examples of the preprocessed and filted geo-electric field data for one month. The unit of the horizontal axis is time, and ‘t/d’ means ‘time/day’. The applied processing method is plotted in the figures.

As a retrospective study, we apply the LURR approach to the geo-electric field near the Yutian *M*_s_ 6.4 earthquake in the Xinjiang region of northwest of china compared with traditional LURR based on Benioff strain release. The raw data of geo-electric field in Hotan observatory were displayed in Fig. 3, which are prepared for LURR calculation. Before calculation of LURR, the geo electric field data are processed by three steps. (1) To remove the pulse data from the geo-electric field time sequences. (2) To linearly interpolate values to the missing data in the geo-electric field sequences. To conduct 8 ~ 24 h bandpass felting on the data to remove component unrelated to the earth tides. Figure 4 shows some examples of the above preprocessed and filtered geo-electric data.

**Figure 5:**
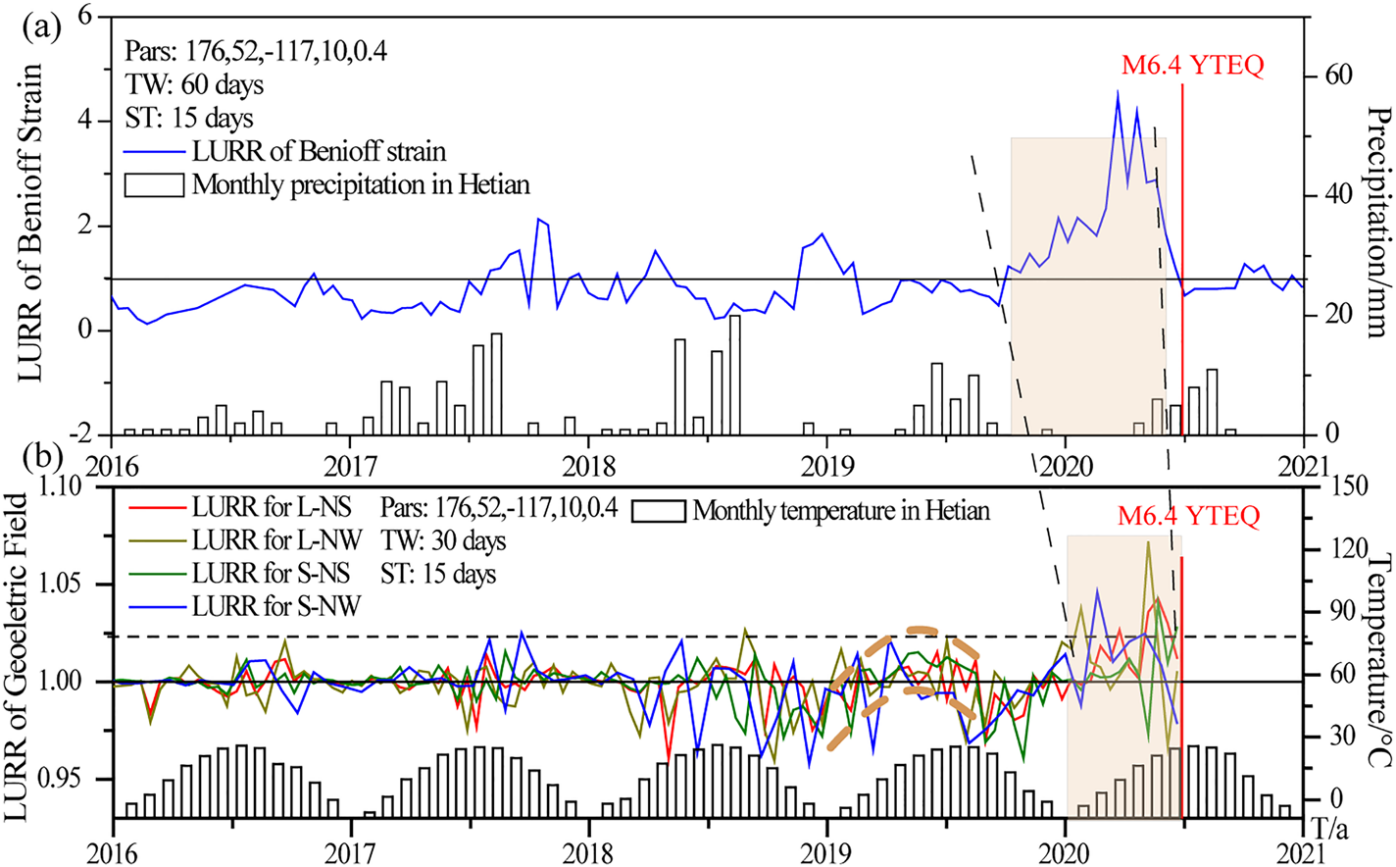
LURR time series curve for Benioff strain (**a**) and geo-electric field (**b**) before the Yutian earthquake. Par is the mechanism of the main shock used for the Coulomb rupture stress calculation, which is: strike, dip, slip, depth, and friction coefficient; TW is the calculation time window; ST is the sliding time window; the red vertical line is the magnitude and time of the Yutian earthquake; the black dashed line indicates the start and end times of anomalous LURR before the occurrence of the Yutian earthquake; the parallel black dotted line is the threshold line of the anomalous LURR. The precipitation and temperature in the Yutian are also shown in the maps. The synchronous changes in early 2019 are highlighted in the maps by brown arc. The unit of the horizontal axis is time, and the ‘t/a’ means ‘time/year’.

Figure 5a shows the sequence curve of LURR anomalies before the Yutian *M*_s_ 6.4 earthquake based on the Benioff strain as response quantity. Data are from the earthquake catalog of CENC. Specific calculation parameters are as follows: the duration of the catalog was from June 1, 2015 to December 31, 2020, and the Benioff strain (*m* = 1/2) for small earthquakes with a magnitude of 0–4.0 within a radius of 300 km from the epicenter was used as the response quantity. Results show that there was no significant anomaly in the Yutian area from 2016 to 2018 (Fig. 5a). The LURR value increased rapidly from October 2019 and reached its peak in March 2020. After 2 months of continuous fluctuation in the high value range, the LURR value decreased rapidly.

For the calculation of LURR of the geo-electric field, we calculated the LURR time series of the electric field in six directions. It was found that four direction finding tracks with long and short pole distances in NS and NW directions, showed common characteristics of synchronous increase before the earthquake (Fig. 5b). The starting time of data was January 2016. LURR values of four directions were fluctuating from the stable value of 1.0, and there was a synchronous but insignificant increase in early 2019. The maximum values of long and short pole distances in the NW direction were 1.025 and 1.026, respectively, and the values of the other two directions were not more than 1.02. LURR timing sequence curves of the four channels showed synchronous changes after December 2019, and the ratio gradually increased at the beginning of 2020. The ratio of the short pole to the NW direction reached the maximum value of 1.049 in March 2020, and the ratio of the other three directions reached the maximum value in May. After that, the amplitude of the anomaly gradually decreased, and the Yutian earthquake occurred during the anomalous return stage of the two tracks (the long pole distance in the NS plotted by red line in Fig. 5b and the short pole distance in the NW plotted by blue line in Fig. 5b).

Figure 5 also displays the monthly precipitation and temperature in the Yutian region where the geo-electric field station is located. During the period of LURR anomalous increase, the two meteorological elements did not change significantly. Therefore, the LURR time series change before the Yutian earthquake should not be caused by the effect of the monthly variation of precipitation and temperature.

## Discussion

### Local stress environment

**Figure 6:**
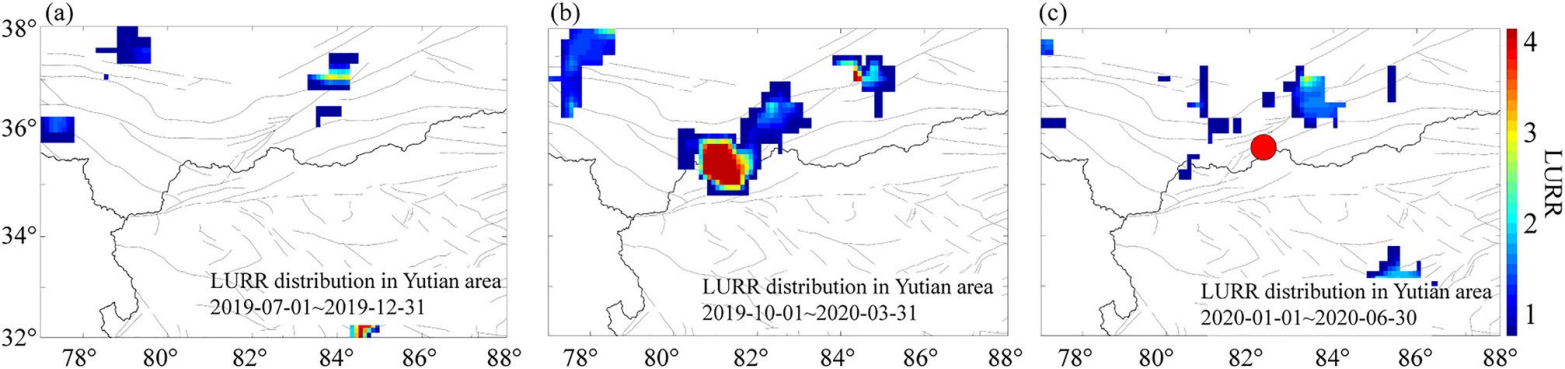
Spatial distribution of LURR anomalies in Xinjiang before the Yutian earthquake. The calculated time window is shown in each of the maps, and the LURR value is given by the color code, and the red dot in (**c**) is the Yutian earthquake. The three figures were created by using the Matlab R2016b (https://www.mathworks.com/products/matlab.html, accessed on 27 September 2022).

In Fig. 6, the spatial distribution of LURR anomalies at three different time periods before the Yutian earthquake was calculated to detect those regions with high stress accumulation. The figures are derived by evaluating LURR of Benioff strain of the *M*_s_ ≤ 4.0 earthquakes (data from the China Earthquake Networks Center catalog). The time window is 0.5 year and the spatial window 80 km radius which slide at a step of 0.25degree in both longitude and latitude. The internal friction coefficient used to evaluate *CFS* is 0.4 and the value of LURR greater than 1.0 is plotted at the center location by using the 2D bilinear interpolation of Matlab.

The results showed that there was no significant anomaly in Yutian region in the second half of 2019 (Fig. 6a), but the anomaly increased significantly in March 2020. It is clear that the spatial distribution of LURR anomaly expanded and the amplitude was also greatly enhanced, especially in the area around the future epicenter where the LURR values exceeded 4 (Fig. 6b). Since then, the LURR values in this region began to weaken before the Yutian earthquake (Fig. 6c).

**Figure 7:**
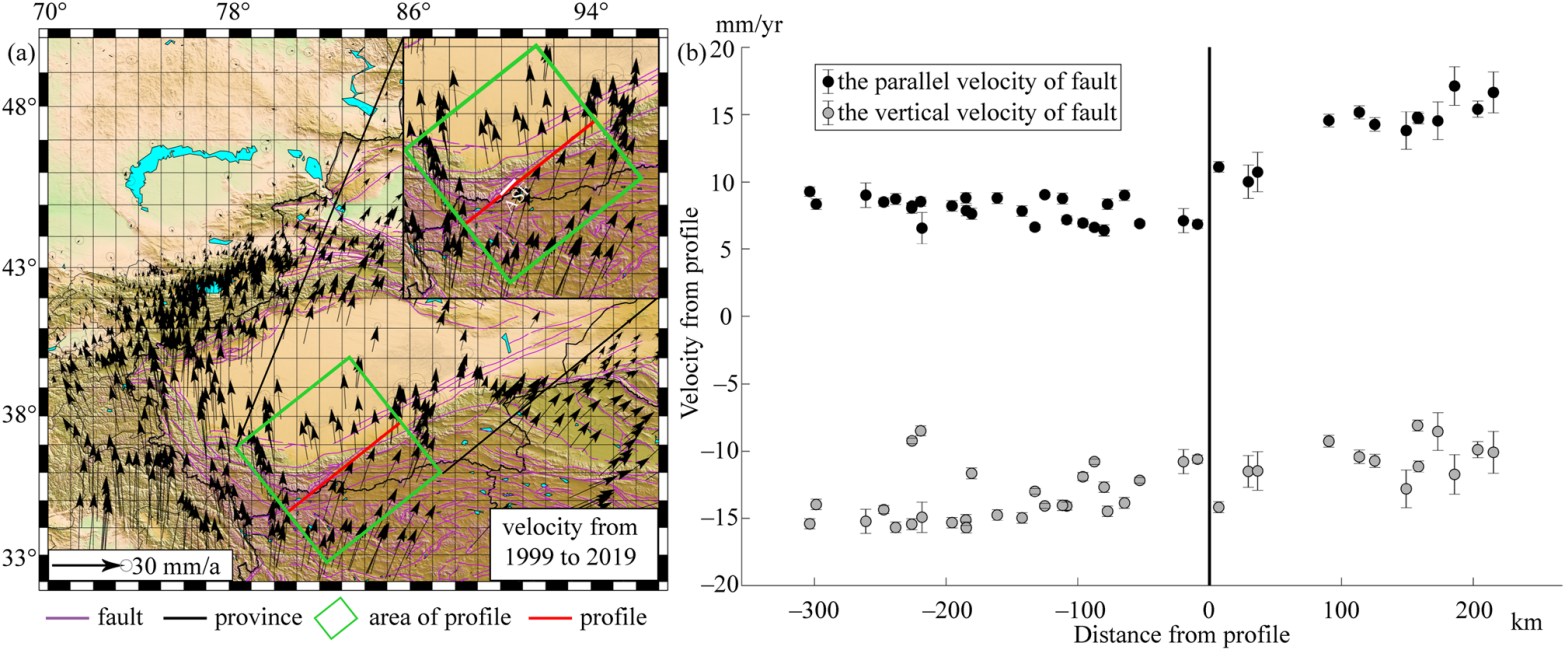
GPS velocity fields before the occurrences of earthquakes on the Ashikule fault zone (**a**) and the N 50°E velocity profile (**b**). Velocity components eastward and northward are designated positive; the white line in the upper panel on the left is the ASF (Ashikule fault). The Fig. 7a was generated by using the Generic Mapping Tools (GMT) 4 (https://www.generic-mapping-tools.org/download/, accessed on 27 September 2022).

In order to evidence that stress accumulation in Yutian earthquake leads to the change of LURR, we constructed a profile. We constructed a GPS profile along the direction of the Ashikule fault using GPS data from 1999 to 2019 to verify the motion properties of the extensional fault area at the southwest end of the Altun Tagh fault zone (Fig. 7a). Calculated data were obtained from the First Monitoring Center of the China Earthquake Administration. Velocity field was calculated using quasi-observational combination analysis^26^, GNSS (global navigation satellite system) at MIT (GAMIT)/Global Kalman filter (GLOBK)^27–29^ of the GPS velocity field associated with mainland China. Results show that the directions of the GPS velocity field within 200 km in the south and 300 km in the north of the profile are in NNE and NNW or N directions, respectively (Fig. 7b). The average velocities on both sides of the section are also significantly different. The NW–SE tensile state of the NE trending rupture fault is dominant, and the slip rates on north and south sides of the fault are approximately 9 and 16 mm/a, respectively, probably because of the northward subduction extrusion of the Southern Indian plate, the obstruction of the Tarim block in the north, and the extrusion of the western Kunlun thrust fault.

### Geodetic data

**Figure 8:**
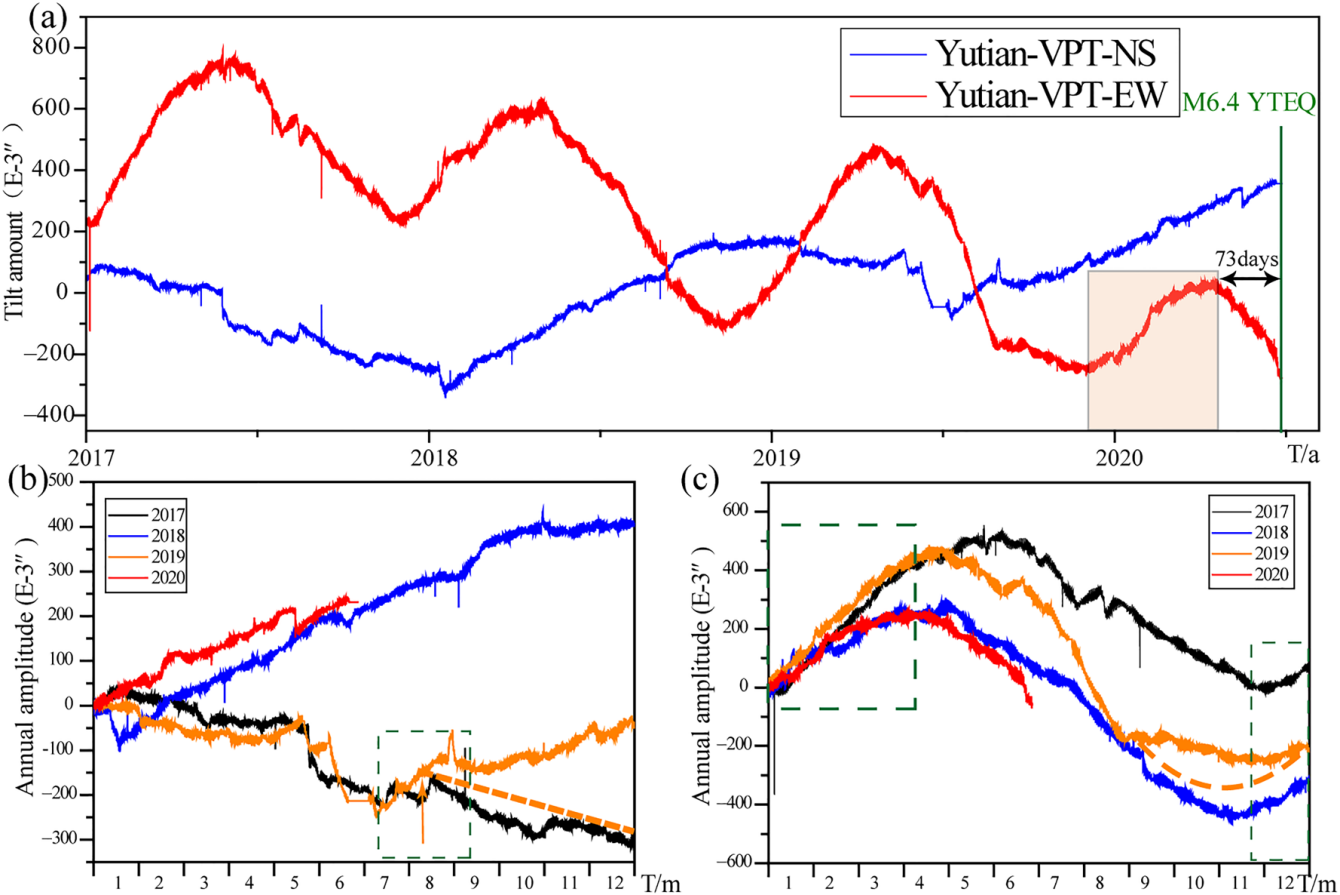
Plots of the vertical pendulum tilt meter recorded at the Yutian Observatory: (**a**) blue and red curves indicate N–S and E–W daily data of the vertical pendulum tilt meter, respectively. The green vertical line is the magnitude and time of the Yutian earthquake; the orange box shows the period of anomalous annual variation (**b**) annual variation of the N–S direction from January 1, 2017 to June 26, 2020; (**c**) annual variation of the E–W direction from January 1, 2017 to June 26, 2020. The turning change of NS component (**b**) in Agust 2019 was caused by the man-made transformation. The yellow dotted line (**b**) represents the previous trend movement from Agust and December. The yellow dotted line of EW component (**c**) represents the likely normal annual trend at the end of 2019. The unit of the horizontal axis is time, and the ‘T/a’ and ‘T/m’ means ‘time/year’ and ‘time/month’ respectively.

This stress state change in the crust calculated by LURR may also be recorded by the near geodetic data in the same time periods (Fig. 8). The vertical pendulum tilt observation of the Yutian Seismic station is located in a cave in Aqiang Township, Yutian County, Xinjiang Uygur Autonomous Region (Fig. 2) with geographic coordinates of 36.4°N and 81.9°E and an elevation of 1200 m. The site consists of clastic carboniferous metamorphic rock and marble. The observation site is located at the intersection of the eastern side of the West Kunlun fold belt and the Tarim Basin. The Pulu fault, which was active in early Quaternary in the NE direction, is distributed approximately 8 km to the north of the station. Approximately 40 km to the east of the station is the Altun Tagh fault zone, which is NE-trending and active in Holocene. Approximately 47 km to the south is the Karakax fault zone, which is NE-trending, and active in Holocene. The station was built in October 2009, within a room in the cave. The cave is 30 m long and it is powered by solar energy. Formal observation began in November 2016.

As shown in the daily mean curve (Fig. 8a), the EW component observed by the tilting of the Yutian vertical pendulum tilt was usually in an E-dip state from mid-September to mid-April of the following year. The time of the E-dip phase was from November 27th, 2019 to April 14th, 2020, and it is the same period as in previous years; however, the annual variation in this period is significantly reduced compared to those of same periods in previous years (Fig. 8c). There was no environmental interference in the site during the period of observation. Comparative statistics shows that the annual variation from 2019 to 2020 is 0.264″ (Table 2), which is significantly smaller than the annual variation during the same period in previous years, and hence, there is an annual variation anomaly. The Yutian *M*_s_ 6.4 earthquake occurred 73 days after the end of the anomaly (Fig. 8a).

**Table 2:** Statistics of the annual variation of the EW component of the vertical pendulum tilt.

Year	2016–2017	2017–2018	2018–2019	2019–2020
Amplitude of annual variation /10^−3^″	712	416	604	264

Anomalous changes in Yutian vertical pendulum near the epicenter of the Yutian earthquake two months before the earthquake reflect the instability and rupture of the fault zone in this region. Previous studies showed that the Altun Tagh, Kunlun, and Karakax fault zones and other strike-slip faults were formed in the northern boundary of the Qinghai–Tibet Plateau due to the eastward movement of the Qinghai–Tibet Plateau block and the obstruction of the Tarim Basin^30–32^. The GPS velocity of the Altun Tagh fault gradually decreases from west to east^33^. The GPS velocity of the western segment is relatively small, its compression is the largest, and surface deformation mainly occurs in the south. However, in the north, due to the stable Tarim block, there are few surface shape variables. As shown in Fig. 7b, the south side of the Yutian earthquake’s seismogenic fault moves eastward at a speed of approximately 3 mm/a. Approximately two months before the earthquake, anomalous deformation observed in the northwest direction of the earthquake shows that Altun Tagh and Karakax faults show eastward movement, and the rate of movement of the former is higher than that of the latter. The instability and loosening of the fault junction had occurred two months before the earthquake because of the long-term pull action and continuous accumulation of stress at the fault junction, and the Karakax fault had temporarily escaped from the eastward extrusion of the Qinghai–Tibet Plateau. The westward movement occurred under the influence of the Tarim Basin, resulting in the tensile characteristics of the Ashikule fault. When the accumulation of tensile stress exceeded the critical rupture state, the Yutian *M*_s_ 6.4 earthquake occurred in 2020.

### Correlation between Benioff strain and geoelectric LURR

LURR models can be used to detect the variations of crustal stress state in the source area before an earthquake occurs. LURR in this paper is evaluated based on the dynamic evolution law of rock constitutive relationship. All geophysical quantities that can reflect the medium instability process in a seismogenic area can be regarded as response quantities. LURR theory is based on the triggering mechanism of tidal stress. Since tidal stress is far lower than tectonic stress, it can only trigger but not create earthquakes. When the tectonic stress is low, small changes of tidal stress are difficult to trigger earthquakes, which mean the crustal medium is in a stable state, hence, LURR < 1. However, when the tectonic stress accumulates to a high level, any small increase of stress, such as tidal stress, may lead to the occurrence of small earthquakes, resulting in differences in the release of Benioff strain between loading and unloading stages, thus, LURR > 1. The Benioff strain response calculation method based on the LURR has a better continuity compared to geophysical observation data. Small changes in LURR volatility in the time curve can accurately identify the critical state before the occurrence of an earthquake, when the rock medium is in an elastic stage (steady state) and the expansion is full in the seismic zone.

**Figure 9:**
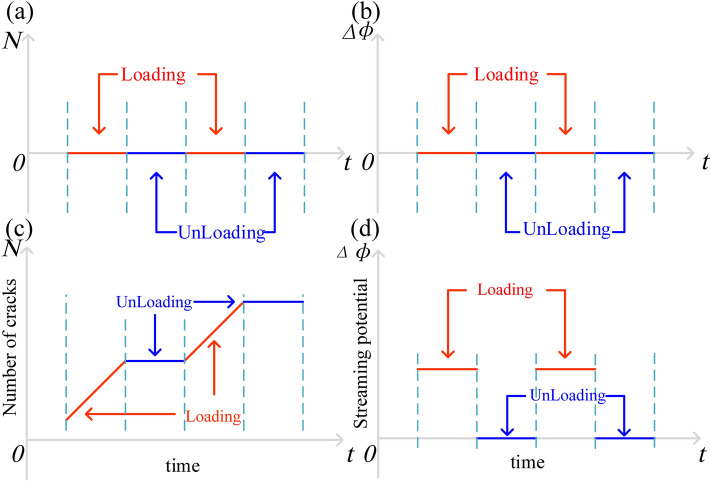
Rupture number and potential difference in steady and damage states during the loading and unloading process; (**a**) and (**b**) are changes in fracture number and flow potential in the elastic stage, respectively; (**c**) and (**d**) are changes in fracture number and flow potential in the dilatant stage, respectively.

LURR of geo-electric field is based on the premise that the expansion of rock volume caused by the cracks generated in critical state of earthquake can change geo-electric field near the focal area. In particular, new cracks were not generated in the solid medium during the loading and unloading process, the number of cracks remained unchanged (Fig. 9a), and the migration of fluid in the rock mass was not affected, and hence, the change in flow potential was very small (Fig. 9b). In addition, the change in the geo-electric field LURR time curve and the LURR value were close to 1.0. As stress accumulation reaches a high level, the rock enters the yield stage (instability state), and according to the Kaiser effect^34^, the number of cracks generated in the rock experiment at this stage was significantly higher than that in the unloading stage (Fig. 9c). At the loading stage, rock dilatation and the generation of microcracks lead to changes in fluid migration, resulting in filtration potential (Fig. 9d). Therefore, the generated local electric field will cause the LURR value to increase gradually and deviate from the stable value of 1^35,36^.

Our calculation results display that the LURR has very significant prediction value. As shown in Fig. 5, the Benioff strain LURR anomaly near the epicenter began to rise rapidly around October 2019 and reach the peak in March 2020. At this time, the LURR anomaly of the Hotan geoelectric field, which is 290 km away from the epicenter, also changed simultaneously, but not significantly. After that, it began to rise rapidly around December 2019, reach the peak in 1 to 3 months before the mainshock. This phenomenon indicates that the change in electric field is related to fracture propagation and fluid infiltration during earthquake preparation^18,19,37^. Anomalous changes in the electric field often occur in the late seismogenic stage^38^, and hence, the peak value of electric field anomaly may be later than that of the LURR of the Benioff strain as the response quantity.

## Conclusion

By calculating the ratio between the geo-electric field during the loading and unloading phase induced by earth tides, we found that this approach might detect the electromechanical coupling process preceding the occurrence of a large earthquake. For Yutian 6.4 earthquake in Xinjiang region, the LURR value of four geo-electric field observation channels exceed 1.02 and the anomalies can last several months. These changes might be caused by infiltration of fluids in the pre-seismic dilatancy. Comparing the LURR time series derived from the Benioff strain and geo-electric data, the significant electromechanical coupling process can be observed prior to the final mainshock. Combination of geodetic time series, we can eliminate more interference information for detecting the upcoming large earthquake. Although more data testing is needed to approve the approach, the results of this paper show that geo-electric field could be used as a feasible physical field to evaluate seismic hazard.

## Data Availability

The earthquake catalog was provided by China Earthquake Networks Center. GPS data were derived from the First Monitoring Center of the China Earthquake Administration. The moment tensors of Yutian *M*_s_ 6.4 were obtained from GCMT (https://www.globalcmt.org/), accessed on 27 Dec 2021. The data has been uploaded to the Mendeley data, which has been published on the repository (https://data.mendeley.com/datasets/4cj2my2z44/1).
